# STRATEGIES USED BY STAFF TO PROVIDE PERSON-CENTERED DEMENTIA CARE: A COMPARISON BY LENGTH OF TENURE

**DOI:** 10.1093/geroni/igae098.0571

**Published:** 2024-12-31

**Authors:** Yoon Chung Kim, Michael Lepore, Nancy Kusmaul, Sarah Holmes

**Affiliations:** University of Maryland Baltimore, Baltimore, Maryland, United States; University of Massachusetts Amherst, Amherst, Massachusetts, United States; University of Maryland Baltimore County, Baltimore, Maryland, United States; University of Maryland School of Nursing, Baltimore, Maryland, United States

## Abstract

Approximately half of nursing home residents and 42% of assisted living residents have a diagnosis of dementia. The quality of care that staff provide to long-term care (LTC) residents living with dementia (RLWD) is vital for their well-being. However, questions remain about what strategies staff use to support person-centered dementia care and whether differences exist by length of tenure. This descriptive, qualitative study sought to identify strategies used by direct care workers to provide person-centered dementia care and compare strategies by length of tenure. The study included in-depth semi-structured interviews with a purposive sample of 20 direct care workers in four low-resource LTC settings in two states. Interviews were audio recorded, transcribed, and thematically analyzed in NVivo14. Staff reported a mean length of tenure in LTC of 8.97 years (SD= 5.92, range= 1.5-21 years). Common strategies recommended by direct care workers for providing person-centered dementia care included validating and recognizing residents’ emotional needs, building trust and rapport, involving families, honoring preferences and life histories, knowing triggers or past traumas, and engaging residents in individualized activities. Participants emphasized the importance of dementia training for providing care to RLWD. There were no differences in strategies observed between longer and shorter tenured direct care workers, but those with longer tenure expressed more emotional detachment regarding resident care, whereas shorter tenured staff expressed more emotional attachment in resident care. Findings highlight how staff with shorter and longer tenure experience care differently and inform future work to promote high-quality care for RLWD in LTC settings.

